# Supplementary material for: Mixed-methods study of university students’ perceptions of COVID-19 and media consumption from March 2020 –April 2022
Source: PLOS Glob Public Health. 2024 Jul 17;4(7):e0003251. doi: 10.1371/journal.pgph.0003251 (PMC11253943; doi:10.1371/journal.pgph.0003251)
Supplement: S1 Text — (DOCX) [file pgph.0003251.s001.docx]

**Survey Guide Round 1: March/April 2020**

1. What is your age? (write in box)
2. To which gender identity do you most identify?
   1. Male
   2. Female
   3. Gender variant/non-conforming
   4. Prefer not to answer
   5. Prefer to self describe (write in box below)
3. How do you describe your ethnicity? (box provided to write in answer)
4. What is your year of study at the University of Toronto?
   1. Undergraduate year 1
   2. Undergraduate year 2
   3. Undergraduate year 3
   4. Undergraduate year 4
   5. Undergraduate year 5
   6. Undergraduate year other
   7. Graduate
5. What is your program of study at the University of Toronto? (write in box)
6. What is the location of your permanent residence?
   1. Greater Toronto Area (GTA)
   2. Southwestern Ontario (other than the GTA)
   3. Northern Ontario
   4. Quebec
   5. Eastern Canadian provinces
   6. Western Canadian provinces
   7. Eastern Ontario
   8. Other (write in below)
7. What is your household income (if you support yourself) or your family’s annual household income, if they provide more than 50% of your income support (CAD)?
   1. Less than $24 999
   2. $25 000 to $49 999
   3. $50 000 to $74 999
   4. $75 000 to $99 999
   5. $100 000 to $124 999
   6. $125 000 to $149 999
   7. Greater than $150 000
8. Have you been personally affected by the current COVID-19 outbreak through the illness of yourself or an immediate family member?
   1. Yes
   2. No
9. Do you have regular access to social media (e.g., Instagram, Facebook, Twitter, TikTok) as a news source?
   1. Yes (if yes, move to Questions 10-12)
   2. No (if no, move to Question 13)
10. In a typical week, how much time per DAY (in minutes) do you spend using social media for general use? (write in box)
11. In a typical week, how much time per DAY (in minutes) do you spend using social media as a source for COVID-19 health-related information?
12. How successful do you feel social media is in bringing clear, concise, and unbiased information about the COVID-19 outbreak?
    1. 7-point Likert scale (1: unsuccessful, 7: successful)
13. Do you have regular access to internet news sources (e.g., CBC, BBC, CNN, Globe and Mail) as a news source?)
    1. Yes (if yes, move to Questions 14-16)
    2. No (if no, move to Question 17)
14. In a typical week, how much time per DAY (in minutes) do you spend using internet news sources for general use? (write in box)
15. In a typical week, how much time per DAY (in minutes) do you spend using internet news sources as a source for COVID-19 health-related information? (write in box)
16. How successful do you feel internet news sources are in bringing clear, concise, and unbiased information about the COVID-19 outbreak?
    1. 7-point Likert scale (1: unsuccessful, 7: successful)
17. Do you have regular access to the radio as a news source?
    1. Yes (if yes, move to Questions 18-20)
    2. No (if no, move to Question 21)
18. In a typical week, how much time per DAY (in minutes) do you spend using the radio for general use? (write in box)
19. In a typical week, how much time per DAY (in minutes) do you spend using the radio as a source for COVID-19 health-related information? (write in box)
20. How successful do you feel the radio is in bringing clear, concise, and unbiased information about the COVID-19 outbreak?
    1. 7-point Likert scale (1: unsuccessful, 7: successful)
21. Do you have regular access to television as a news source?
    1. Yes (if yes, move to Questions 22-24)
    2. No (if no, move to Question 25)
22. In a typical week, how much time per DAY (in minutes) do you spend using television for general use? (write in box)
23. In a typical week, how much time per DAY (in minutes) do you spend using television as a source for COVID-19 health-related information? (write in box)
24. How successful do you feel television is in bringing clear, concise, and unbiased information about the COVID-19 outbreak?
    1. 7-point Likert scale (1: unsuccessful, 7: successful)
25. Do you have regular access to magazines as a news source?
    1. Yes (if yes, move to Questions 26-28)
    2. No (if no, move to Question 29)
26. In a typical week, how much time per DAY (in minutes) do you spend using magazines for general use? (write in box)
27. In a typical week, how much time per DAY (in minutes) do you spend using magazines as a source for COVID-19 health-related information? (write in box)
28. How successful do you feel magazines are in bringing clear, concise, and unbiased information about the COVID-19 outbreak?
    1. 7-point Likert scale (1: unsuccessful, 7: successful)
29. How anxious or fearful of acquiring COVID-19 are you after hearing or reading a news report updating the outbreak?
    1. 7-point Likert scale (1: not fearful/anxious, 7: very fearful/anxious)
30. How much time does the media spend covering the COVID-19 outbreak?
    1. 7-point Likert scale (1: not enough, 7: too much)
31. How severe a threat is COVID-19?
    1. 7-point Likert scale (1: not severe, 7: very severe)
32. Do you think you are at risk of contracting COVID-19? Explain why or why not.
33. Which form of media made you feel the most anxiety and fear about becoming infected with COVID-19?
    1. Social media
    2. Internet news source
    3. Radio
    4. Television
    5. Magazines
    6. Other (write in below)
34. What do you know about the origin of COVID-19?
35. In your opinion, is COVID-19 worse than the Spanish flu?
    1. Yes
    2. No
    3. About the Same
    4. I Don’t Know
    5. Expand on your choice in box below
36. In your opinion, is COVID-19 worse than SARS?
    1. Yes
    2. No
    3. About the Same
    4. I Don’t Know
    5. Expand on your choice in box below
37. In your opinion, is COVID-19 worse than swine flu (H1N1)?
    1. Yes
    2. No
    3. About the Same
    4. I Don’t Know
    5. Expand on your choice in box below
38. Has the news coverage of COVID-19 prevented you from travelling or otherwise influenced a decision about travelling?
    1. Yes
    2. No
39. Has the news coverage of COVID-19 influenced your day-to-day behaviour? (Choose all that apply)
    1. I wear a mask some or all of the time
    2. I am washing my hands more
    3. I am using hand sanitizer
    4. I am social distancing
    5. I have bought extra food/supplies
    6. I have gone to or plan to go to the doctor for help/advice
    7. I have gone to or plan to go to the hospital for help/advice
    8. I have called or plan to call public health for help/advice
    9. Other (write in box below)
    10. No
40. How have you employed social distancing in your life? Please explain how this has affected your daily activities (write in box)
41. What are your impressions of the current news media stories about COVID-19? (write in box)
42. What do you think are the main factors influencing the spread of COVID-19? (write in box)
43. What do you think the Canadian government could be doing to reduce the risk of COVID-19? (write in box)
44. What do you remember about the SARS outbreak in 2003? (write in box)
45. Is there anything else you would like to say about the COVID-19 outbreak or your response to it that we did not ask? (write in box)
46. As part of our research we are looking for participants to be interviewed about their perceptions of COVID-19 over videoconferencing. All interviews will be confidential. If you are interested in receiving more information about the interview, please write your email into the box below and we will contact you. Providing your email does NOT mean you must participate.
47. Thank you for participating in our survey! If you would like to be entered into a draw for one of three $50 gift cards please enter your email below. Please press “Submit” below to complete the survey.

**Survey Guide Round 2: June/July 2020**

1. What is your age? (write in box)
2. To which gender identity do you most identify?
   1. Male
   2. Female
   3. Gender variant/non-conforming
   4. Prefer not to answer
   5. Prefer to self describe (write in box below)
3. How do you describe your ethnicity? (box provided to write in answer)
4. What is your year of study at the University of Toronto?
   1. Undergraduate year 1
   2. Undergraduate year 2
   3. Undergraduate year 3
   4. Undergraduate year 4
   5. Undergraduate year 5
   6. Undergraduate year other
   7. Graduate
5. What is your program of study at the University of Toronto? (write in box)
6. What is the location of your permanent residence?
   1. Greater Toronto Area (GTA)
   2. Southwestern Ontario (other than the GTA)
   3. Northern Ontario
   4. Quebec
   5. Eastern Canadian provinces
   6. Western Canadian provinces
   7. Eastern Ontario
   8. Other (write in below)
7. What is your household income (if you support yourself) or your family’s annual household income, if they provide more than 50% of your income support (CAD)?
   1. Less than $24 999
   2. $25 000 to $49 999
   3. $50 000 to $74 999
   4. $75 000 to $99 999
   5. $100 000 to $124 999
   6. $125 000 to $149 999
   7. Greater than $150 000
8. Have you been personally affected by the current COVID-19 outbreak through the illness of yourself or an immediate family member?
   1. Yes
   2. No
9. Do you have regular access to social media (e.g., Instagram, Facebook, Twitter, TikTok) as a news source?
   1. Yes (if yes, move to Questions 10-12)
   2. No (if no, move to Question 13)
10. In a typical week, how much time per DAY (in minutes) do you spend using social media for general use? (write in box)
11. In a typical week, how much time per DAY (in minutes) do you spend using social media as a source for COVID-19 health-related information?
12. How successful do you feel social media is in bringing clear, concise, and unbiased information about the COVID-19 outbreak?
    1. 7-point Likert scale (1: unsuccessful, 7: successful)
13. Do you have regular access to internet news sources (e.g., CBC, BBC, CNN, Globe and Mail) as a news source?)
    1. Yes (if yes, move to Questions 14-16)
    2. No (if no, move to Question 17)
14. In a typical week, how much time per DAY (in minutes) do you spend using internet news sources for general use? (write in box)
15. In a typical week, how much time per DAY (in minutes) do you spend using internet news sources as a source for COVID-19 health-related information? (write in box)
16. How successful do you feel internet news sources are in bringing clear, concise, and unbiased information about the COVID-19 outbreak?
    1. 7-point Likert scale (1: unsuccessful, 7: successful)
17. Do you have regular access to the radio as a news source?
    1. Yes (if yes, move to Questions 18-20)
    2. No (if no, move to Question 21)
18. In a typical week, how much time per DAY (in minutes) do you spend using the radio for general use? (write in box)
19. In a typical week, how much time per DAY (in minutes) do you spend using the radio as a source for COVID-19 health-related information? (write in box)
20. How successful do you feel the radio is in bringing clear, concise, and unbiased information about the COVID-19 outbreak?
    1. 7-point Likert scale (1: unsuccessful, 7: successful)
21. Do you have regular access to television as a news source?
    1. Yes (if yes, move to Questions 22-24)
    2. No (if no, move to Question 25)
22. In a typical week, how much time per DAY (in minutes) do you spend using television for general use? (write in box)
23. In a typical week, how much time per DAY (in minutes) do you spend using television as a source for COVID-19 health-related information? (write in box)
24. How successful do you feel television is in bringing clear, concise, and unbiased information about the COVID-19 outbreak?
    1. 7-point Likert scale (1: unsuccessful, 7: successful)
25. Do you have regular access to magazines as a news source?
    1. Yes (if yes, move to Questions 26-28)
    2. No (if no, move to Question 29)
26. In a typical week, how much time per DAY (in minutes) do you spend using magazines for general use? (write in box)
27. In a typical week, how much time per DAY (in minutes) do you spend using magazines as a source for COVID-19 health-related information? (write in box)
28. How successful do you feel magazines are in bringing clear, concise, and unbiased information about the COVID-19 outbreak?
    1. 7-point Likert scale (1: unsuccessful, 7: successful)
29. How anxious or fearful of acquiring COVID-19 are you after hearing or reading a news report updating the outbreak?
    1. 7-point Likert scale (1: not fearful/anxious, 7: very fearful/anxious)
30. How much time does the media spend covering the COVID-19 outbreak?
    1. 7-point Likert scale (1: not enough, 7: too much)
31. How severe a threat is COVID-19?
    1. 7-point Likert scale (1: not severe, 7: very severe)
32. Do you think you are at risk of contracting COVID-19?
    1. Yes
    2. No
33. Why do you think you are or are not at risk of contracting COVID-19? (write in box)
34. Which form of media made you feel the most anxiety and fear about becoming infected with COVID-19?
    1. Social media
    2. Internet news source
    3. Radio
    4. Television
    5. Magazines
    6. Other (write in below)
35. Has the news coverage of COVID-19 influenced your day-to-day behaviour? (Choose all that apply)
    1. No change
    2. Cancelled or changed travel plans
    3. I wear a mask some or all of the time
    4. I am washing my hands more
    5. I am using hand sanitizer
    6. I am social distancing
    7. I am self isolating
    8. I am cleaning more
    9. I have bought extra food/supplies
    10. I have gone to or plan to go to the doctor for help/advice
    11. I have gone to or plan to go to the hospital for help/advice
    12. I have called public health
    13. I have been tested for COVID-19
    14. Other (write in box below)
36. What do you think are the main factors influencing the spread of COVID-19? (write in box)
37. What effects have the government-mandated social distancing and non-essential closures had on your life (employment, financial, social, etc.)? (write in box)
38. How are the social distancing rules and the pandemic in general affecting your mental health? (write in box)
39. What strategies, support systems, or programs for mental health or otherwise (economic, social) would you find beneficial at this time (what could the local, provincial, or federal government be doing to help you)? (write in box)
40. What is your opinion of the current plan to re-open your province/city/town? What do you feel is being done well and what could be done differently? (write in box)
41. Have you experienced, heard of, or witnessed any racism towards Asian Canadians during the pandemic? If so, what have you seen/heard/read? (write in box)
42. If a vaccine for COVID-19 were to become available, would you want to get it?
    1. Yes
    2. No
    3. Expand on your answer (write in box)
43. If a vaccine trial was run in Canada, would you volunteer to be part of the trial?
    1. Yes
    2. No
    3. Expand on your answer (write in box)
44. Is there anything else you would like to say about the COVID-19 outbreak or your response that we did not ask? (write in box)
45. Did you participate in the first round of this survey (March/April 2020)?
    1. Yes
    2. No
46. As part of our research we are looking for participants to be interviewed about their perceptions of COVID-19 over videoconferencing. All interviews will be confidential. If you are interested in receiving more information about the interview, please write your email into the box below and we will contact you. Providing your email does NOT mean you must participate.
47. Thank you for participating in our survey! If you would like to be entered into a draw for one of three $50 gift cards please enter your email below. Please press “Submit” below to complete the survey.

**Survey Guide Round 3: September/October 2020**

1. What is your age? (write in box)
2. To which gender identity do you most identify?
   1. Male
   2. Female
   3. Gender variant/non-conforming
   4. Prefer not to answer
   5. Prefer to self describe (write in box below)
3. How do you describe your ethnicity? (write in box)
4. What is your year of study at the University of Toronto?
   1. Undergraduate year 1
   2. Undergraduate year 2
   3. Undergraduate year 3
   4. Undergraduate year 4
   5. Undergraduate year 5
   6. Undergraduate year other
   7. Graduate
5. What is your program of study at the University of Toronto? (write in box)
6. What is the location of your permanent residence?
   1. Greater Toronto Area (GTA)
   2. Southwestern Ontario (other than the GTA)
   3. Northern Ontario
   4. Quebec
   5. Eastern Canadian provinces
   6. Western Canadian provinces
   7. Eastern Ontario
   8. Other (write in below)
7. What is your household income (if you support yourself) or your family’s annual household income, if they provide more than 50% of your income support (CAD)?
   1. Less than $24 999
   2. $25 000 to $49 999
   3. $50 000 to $74 999
   4. $75 000 to $99 999
   5. $100 000 to $124 999
   6. $125 000 to $149 999
   7. Greater than $150 000
8. Have you been personally affected by the current COVID-19 outbreak through the illness of yourself or an immediate family member?
   1. Yes
   2. No
9. Do you have regular access to social media (e.g., Instagram, Facebook, Twitter, TikTok) as a news source?
   1. Yes (if yes, move to Questions 10-12)
   2. No (if no, move to Question 13)
10. In a typical week, how much time per DAY (in minutes) do you spend using social media for general use? (write in box)
11. In a typical week, how much time per DAY (in minutes) do you spend using social media as a source for COVID-19 health-related information?
12. How successful do you feel social media is in bringing clear, concise, and unbiased information about the COVID-19 outbreak?
    1. 7-point Likert scale (1: unsuccessful, 7: successful)
13. Do you have regular access to internet news sources (e.g., CBC, BBC, CNN, Globe and Mail) as a news source?)
    1. Yes (if yes, move to Questions 14-16)
    2. No (if no, move to Question 17)
14. In a typical week, how much time per DAY (in minutes) do you spend using internet news sources for general use? (write in box)
15. In a typical week, how much time per DAY (in minutes) do you spend using internet news sources as a source for COVID-19 health-related information? (write in box)
16. How successful do you feel internet news sources are in bringing clear, concise, and unbiased information about the COVID-19 outbreak?
    1. 7-point Likert scale (1: unsuccessful, 7: successful)
17. Do you have regular access to the radio as a news source?
    1. Yes (if yes, move to Questions 18-20)
    2. No (if no, move to Question 21)
18. In a typical week, how much time per DAY (in minutes) do you spend using the radio for general use? (write in box)
19. In a typical week, how much time per DAY (in minutes) do you spend using the radio as a source for COVID-19 health-related information? (write in box)
20. How successful do you feel the radio is in bringing clear, concise, and unbiased information about the COVID-19 outbreak?
    1. 7-point Likert scale (1: unsuccessful, 7: successful)
21. Do you have regular access to television as a news source?
    1. Yes (if yes, move to Questions 22-24)
    2. No (if no, move to Question 25)
22. In a typical week, how much time per DAY (in minutes) do you spend using television for general use? (write in box)
23. In a typical week, how much time per DAY (in minutes) do you spend using television as a source for COVID-19 health-related information? (write in box)
24. How successful do you feel television is in bringing clear, concise, and unbiased information about the COVID-19 outbreak?
    1. 7-point Likert scale (1: unsuccessful, 7: successful)
25. Do you have regular access to magazines as a news source?
    1. Yes (if yes, move to Questions 26-28)
    2. No (if no, move to Question 29)
26. In a typical week, how much time per DAY (in minutes) do you spend using magazines for general use? (write in box)
27. In a typical week, how much time per DAY (in minutes) do you spend using magazines as a source for COVID-19 health-related information? (write in box)
28. How successful do you feel magazines are in bringing clear, concise, and unbiased information about the COVID-19 outbreak?
    1. 7-point Likert scale (1: unsuccessful, 7: successful)
29. How anxious or fearful of acquiring COVID-19 are you after hearing or reading a news report updating the outbreak?
    1. 7-point Likert scale (1: not fearful/anxious, 7: very fearful/anxious)
30. How much time does the media spend covering the COVID-19 outbreak?
    1. 7-point Likert scale (1: not enough, 7: too much)
31. How severe a threat is COVID-19?
    1. 7-point Likert scale (1: not severe, 7: very severe)
32. Do you think you are at risk of contracting COVID-19?
    1. Yes
    2. No
33. Why do you think you are or are not at risk of contracting COVID-19? (write in box)
34. Which form of media made you feel the most anxiety and fear about becoming infected with COVID-19?
    1. Social media
    2. Internet news source
    3. Radio
    4. Television
    5. Magazines
    6. Other (write in below)
35. Has the news coverage of COVID-19 influenced your day-to-day behaviour? (Choose all that apply)
    1. No change
    2. Cancelled or changed travel plans
    3. I wear a mask some or all of the time
    4. I am washing my hands more
    5. I am using hand sanitizer
    6. I am social distancing
    7. I am self isolating
    8. I am cleaning more
    9. I have bought extra food/supplies
    10. I have gone to or plan to go to the doctor for help/advice
    11. I have gone to or plan to go to the hospital for help/advice
    12. I have called public health
    13. I have been tested for COVID-19
    14. I have chosen to get a COVID-19 test
    15. Other (write in box below)
36. What do you think are the main factors influencing the spread of COVID-19? (write in box)
37. What effects have the government-mandated social distancing and non-essential closures had on your life (employment, financial, social, etc.)? (write in box)
38. How are the social distancing rules and the pandemic in general affecting your mental health? (write in box)
39. What strategies, support systems, or programs for mental health or otherwise (economic, social) would you find beneficial at this time (what could the local, provincial, or federal government be doing to help you)? (write in box)
40. What is your opinion of the current plan to re-open your province/city/town? What do you feel is being done well and what could be done differently? (write in box)
41. Have you experienced, heard of, or witnessed any racism towards Asian Canadians during the pandemic? If so, what have you seen/heard/read? (write in box)
42. Do you usually get the seasonal flu vaccine?
    1. Yes (if yes, move to Question 43)
    2. No (if no, move to Question 44)
43. If yes, why? (Select all that apply)
    1. To avoid catching the flu
    2. My doctor recommends that I get it
    3. To avoid illness
    4. It is safe
    5. Worried about becoming seriously ill
    6. I always get the seasonal flu shot
    7. I live with people who are high risk
    8. I am high risk
    9. I am required to because of my job
    10. Other (write in below)
44. If no, why? (Select all that apply)
    1. It will not work
    2. Worried it will cause serious side effects
    3. Worried it will cause bothersome side effects
    4. Worried it will give me the flu
    5. It is not safe
    6. I am not at risk of catching the flu
    7. I don’t know where I would get it
    8. Other (write in below)
45. Did you get the seasonal flu vaccine last year?
    1. Yes
    2. No
    3. Can’t remember
46. Do you plan to get the seasonal flu vaccine this year?
    1. Yes
    2. No
    3. Undecided
47. If a vaccine for COVID-19 were to become available, would you want to get it?
    1. Yes (if yes, move to Question 48)
    2. No (if no, move to Question 49)
48. If yes, why? (Select all that apply)
    1. To avoid catching COVID-19
    2. To avoid illness
    3. It is safe
    4. Worried about becoming seriously ill
    5. COVID-19 is deadlier than the seasonal flu
    6. I always get the seasonal flu shot
    7. I live with people who are high risk
    8. I am high risk
    9. I will be required to because of my job
    10. Other (write in below)
49. If no, why? (Select all that apply)
    1. It will not work
    2. Insufficient testing
    3. Worried it will cause serious side effects
    4. Worried it will cause bothersome side effects
    5. Worried it would give me COVID-19
    6. It is not safe
    7. I am not at risk of catching COVID-19
    8. I don’t know where I would get it
    9. Other (write in below)
50. If a vaccine trial was run in Canada, would you volunteer to be part of the trial?
    1. Yes
    2. No
    3. Expand on your answer (write in box)
51. If your doctor or pharmacist recommended getting the COVID-19 vaccine would that encourage you to get it?
    1. Yes
    2. No
    3. Undecided
52. Did you participate in the first round (March/April 2020) or second round (June/July 2020) of this survey? Select all that apply.
    1. Neither
    2. First round (March/April)
    3. Second round (June/July)
53. As part of our research we are looking for participants to be interviewed about their perceptions of COVID-19 over videoconferencing. All interviews will be confidential. If you are interested in receiving more information about the interview, please write your email into the box below and we will contact you. Providing your email does NOT mean you must participate.
54. Thank you for participating in our survey! If you would like to be entered into a draw for one of three $50 gift cards please enter your email below. Please press “Submit” below to complete the survey.

**Survey Guide Round 4: March/April 2021**

1. What is your age? (write in box)
2. To which gender identity do you most identify?
   1. Male
   2. Female
   3. Gender variant/non-conforming
   4. Prefer not to answer
   5. Prefer to self describe (write in box below)
3. How do you describe your ethnicity? (write in box)
4. What is your year of study at the University of Toronto?
   1. Undergraduate year 1
   2. Undergraduate year 2
   3. Undergraduate year 3
   4. Undergraduate year 4
   5. Undergraduate year 5
   6. Undergraduate year other
   7. Graduate
5. What is your program of study at the University of Toronto? (write in box)
6. What is the location of your permanent residence?
   1. Greater Toronto Area (GTA)
   2. Southwestern Ontario (other than the GTA)
   3. Northern Ontario
   4. Quebec
   5. Eastern Canadian provinces
   6. Western Canadian provinces
   7. Eastern Ontario
   8. Other (write in below)
7. What is your household income (if you support yourself) or your family’s annual household income, if they provide more than 50% of your income support (CAD)?
   1. Less than $24 999
   2. $25 000 to $49 999
   3. $50 000 to $74 999
   4. $75 000 to $99 999
   5. $100 000 to $124 999
   6. $125 000 to $149 999
   7. Greater than $150 000
8. Have you been personally affected by the current COVID-19 outbreak through the illness of yourself or an immediate family member?
   1. Yes
   2. No
9. How severe a threat is COVID-19?
   1. 7-point Likert scale (1: not severe, 7: very severe)
10. How anxious or fearful of acquiring COVID-19 are you after hearing or reading a news report updating the outbreak?
    1. 7-point Likert scale (1: not fearful/anxious, 7: very fearful/anxious)
11. Do you think you are at risk of contracting COVID-19?
    1. Yes
    2. No
12. Why do you think you are or are not at risk of contracting COVID-19? (write in box)
13. Has the news coverage of COVID-19 influenced your day-to-day behaviour? (Choose all that apply)
    1. No change
    2. Cancelled or changed travel plans
    3. I wear a mask some or all of the time
    4. I am washing my hands more
    5. I am using hand sanitizer
    6. I am social distancing
    7. I am self isolating
    8. I am cleaning more
    9. I have bought extra food/supplies
    10. I have gone to or plan to go to the doctor for help/advice
    11. I have gone to or plan to go to the hospital for help/advice
    12. I have called public health
    13. I have been tested for COVID-19
    14. I have chosen to get a COVID-19 test
    15. Other (write in box below)
14. What effects have the government-mandated social distancing and non-essential closures had on your life (employment, financial, social, etc.)? (write in box)
15. How are the social distancing rules and the pandemic in general affecting your mental health? (write in box)
16. What strategies, support systems, or programs for mental health or otherwise (economic, social) would you find beneficial at this time (what could the local, provincial, or federal government be doing to help you)? (write in box)
17. In the last year have you noticed an increase in your use of substances (e.g., alcohol, cannabis, opioids, stimulants, sedatives, hallucinogens)? Why or why not? (write in box)
18. There is discussion of returning to in-person classes in the fall. What university class format would you prefer to have in Fall 2021?
    1. Online only
    2. Face-to-face in person only
    3. Hybrid/blended
    4. Expand on your answer
19. Have you experienced, heard of, or witnessed any racism towards Asian Canadians during the pandemic? If so, what have you seen/heard/read? (write in box)
20. Do you usually get the seasonal flu vaccine?
    1. Yes (if yes, move to Question 43)
    2. No (if no, move to Question 44)
21. If yes, why? (Select all that apply)
    1. To avoid catching the flu
    2. My doctor recommends that I get it
    3. To avoid illness
    4. It is safe
    5. Worried about becoming seriously ill
    6. I always get the seasonal flu shot
    7. I live with people who are high risk
    8. I am high risk
    9. I am required to because of my job
    10. Other (write in below)
22. If no, why? (Select all that apply)
    1. It will not work
    2. Worried it will cause serious side effects
    3. Worried it will cause bothersome side effects
    4. Worried it will give me the flu
    5. It is not safe
    6. I am not at risk of catching the flu
    7. I don’t know where I would get it
    8. Other (write in below)
23. Did you get the seasonal flu vaccine in 2019?
    1. Yes
    2. No
    3. Can’t remember
24. Did you get the seasonal flu vaccine in 2020?
    1. Yes
    2. No
    3. Undecided
25. When a vaccine for COVID-19 becomes available for your age group, would you want to get it?
    1. Yes (if yes, move to Question 26)
    2. No (if no, move to Question 27)
26. If yes, why? (Select all that apply)
    1. To avoid catching COVID-19
    2. To avoid illness
    3. It is safe
    4. Worried about becoming seriously ill
    5. COVID-19 is deadlier than the seasonal flu
    6. I always get the seasonal flu shot
    7. I live with people who are high risk
    8. I am high risk
    9. I will be required to because of my job
    10. Other (write in below)
27. If no, why? (Select all that apply)
    1. It will not work
    2. Insufficient testing
    3. Worried it will cause serious side effects
    4. Worried it will cause bothersome side effects
    5. Worried it would give me COVID-19
    6. It is not safe
    7. I am not at risk of catching COVID-19
    8. I don’t know where I would get it
    9. Other (write in below)
28. Which COVID-19 vaccines (if any) would you be willing to get?
    1. Any vaccine I am offered
    2. None
    3. Pfizer (mRNA)
    4. Moderna (mRNA)
    5. AstraZeneca
    6. Johnson & Johnson
    7. Other
    8. Expand on your answer
29. If your doctor or pharmacist recommended getting the COVID-19 vaccine would that encourage you to get it?
    1. Yes
    2. No
    3. Undecided
30. Do you have regular access to social media (e.g., Instagram, Facebook, Twitter, TikTok) as a news source?
    1. Yes (if yes, move to Questions 10-12)
    2. No (if no, move to Question 13)
31. In a typical week, how much time per DAY (in minutes) do you spend using social media for general use? (write in box)
32. In a typical week, how much time per DAY (in minutes) do you spend using social media as a source for COVID-19 health-related information?
33. How successful do you feel social media is in bringing clear, concise, and unbiased information about the COVID-19 outbreak?
    1. 7-point Likert scale (1: unsuccessful, 7: successful)
34. Do you have regular access to internet news sources (e.g., CBC, BBC, CNN, Globe and Mail) as a news source?)
    1. Yes (if yes, move to Questions 14-16)
    2. No (if no, move to Question 17)
35. In a typical week, how much time per DAY (in minutes) do you spend using internet news sources for general use? (write in box)
36. In a typical week, how much time per DAY (in minutes) do you spend using internet news sources as a source for COVID-19 health-related information? (write in box)
37. How successful do you feel internet news sources are in bringing clear, concise, and unbiased information about the COVID-19 outbreak?
    1. 7-point Likert scale (1: unsuccessful, 7: successful)
38. Do you have regular access to the radio as a news source?
    1. Yes (if yes, move to Questions 18-20)
    2. No (if no, move to Question 21)
39. In a typical week, how much time per DAY (in minutes) do you spend using the radio for general use? (write in box)
40. In a typical week, how much time per DAY (in minutes) do you spend using the radio as a source for COVID-19 health-related information? (write in box)
41. How successful do you feel the radio is in bringing clear, concise, and unbiased information about the COVID-19 outbreak?
    1. 7-point Likert scale (1: unsuccessful, 7: successful)
42. Do you have regular access to television as a news source?
    1. Yes (if yes, move to Questions 22-24)
    2. No (if no, move to Question 25)
43. In a typical week, how much time per DAY (in minutes) do you spend using television for general use? (write in box)
44. In a typical week, how much time per DAY (in minutes) do you spend using television as a source for COVID-19 health-related information? (write in box)
45. How successful do you feel television is in bringing clear, concise, and unbiased information about the COVID-19 outbreak?
    1. 7-point Likert scale (1: unsuccessful, 7: successful)
46. Do you have regular access to magazines as a news source?
    1. Yes (if yes, move to Questions 26-28)
    2. No (if no, move to Question 29)
47. In a typical week, how much time per DAY (in minutes) do you spend using magazines for general use? (write in box)
48. In a typical week, how much time per DAY (in minutes) do you spend using magazines as a source for COVID-19 health-related information? (write in box)
49. How successful do you feel magazines are in bringing clear, concise, and unbiased information about the COVID-19 outbreak?
    1. 7-point Likert scale (1: unsuccessful, 7: successful)
50. How much time does the media spend covering the COVID-19 outbreak?
    1. 7-point Likert scale (1: not enough, 7: too much)
51. Which form of media made you feel the most anxiety and fear about becoming infected with COVID-19?
    1. Social media
    2. Internet news source
    3. Radio
    4. Television
    5. Magazines
    6. Other (write in below)
52. Did you participate in the first round (March/April 2020), second round (June/July 2020), or third round (September/October 2020) of this survey? Select all that apply.
    1. First round (March/April)
    2. Second round (June/July)
    3. Third round (September/October)
    4. None of these
53. As part of our research we are looking for participants to be interviewed about their perceptions of COVID-19 over videoconferencing. All interviews will be confidential. If you are interested in receiving more information about the interview, please write your email into the box below and we will contact you. Providing your email does NOT mean you must participate.
54. Thank you for participating in our survey! If you would like to be entered into a draw for one of three $50 gift cards please enter your email below. Please press “Submit” below to complete the survey.

**Survey Guide Round 5: September/October 2021**

1. What is your age? (write in box)
2. To which gender identity do you most identify?
   1. Male
   2. Female
   3. Gender variant/non-conforming
   4. Prefer not to answer
   5. Prefer to self describe (write in box below)
3. How do you describe your ethnicity? (write in box)
4. What is your year of study at the University of Toronto?
   1. Undergraduate year 1
   2. Undergraduate year 2
   3. Undergraduate year 3
   4. Undergraduate year 4
   5. Undergraduate year 5
   6. Undergraduate year other
   7. Graduate
5. What is your program of study at the University of Toronto? (write in box)
6. What is the location of your permanent residence?
   1. Greater Toronto Area (GTA)
   2. Southwestern Ontario (other than the GTA)
   3. Northern Ontario
   4. Quebec
   5. Eastern Canadian provinces
   6. Western Canadian provinces
   7. Eastern Ontario
   8. Other (write in below)
7. What is your household income (if you support yourself) or your family’s annual household income, if they provide more than 50% of your income support (CAD)?
   1. Less than $24 999
   2. $25 000 to $49 999
   3. $50 000 to $74 999
   4. $75 000 to $99 999
   5. $100 000 to $124 999
   6. $125 000 to $149 999
   7. Greater than $150 000
8. Have you been personally affected by the current COVID-19 outbreak through the illness of yourself or an immediate family member?
   1. Yes
   2. No
9. How severe a threat is COVID-19?
   1. 7-point Likert scale (1: not severe, 7: very severe)
10. How anxious or fearful of acquiring COVID-19 are you after hearing or reading a news report updating the outbreak?
    1. 7-point Likert scale (1: not fearful/anxious, 7: very fearful/anxious)
11. Do you think you are at risk of contracting COVID-19?
    1. Yes
    2. No
12. Why do you think you are or are not at risk of contracting COVID-19? (write in box)
13. Has the news coverage of COVID-19 influenced your day-to-day behaviour? (Choose all that apply)
    1. No change
    2. Cancelled or changed travel plans
    3. I wear a mask some or all of the time
    4. I am washing my hands more
    5. I am using hand sanitizer
    6. I am social distancing
    7. I am self isolating
    8. I am cleaning more
    9. I have bought extra food/supplies
    10. I have gone to or plan to go to the doctor for help/advice
    11. I have gone to or plan to go to the hospital for help/advice
    12. I have called public health
    13. I have been tested for COVID-19
    14. I have chosen to get a COVID-19 test
    15. Other (write in box below)
14. What effects have the government-mandated social distancing and non-essential closures had on your life (employment, financial, social, etc.)? (write in box)
15. How are the social distancing rules and the pandemic in general affecting your mental health? (write in box)
16. What strategies, support systems, or programs for mental health or otherwise (economic, social) would you find beneficial at this time (what could the local, provincial, or federal government be doing to help you)? (write in box)
17. In the last year have you noticed an increase in your use of substances (e.g., alcohol, cannabis, opioids, stimulants, sedatives, hallucinogens)? Why or why not? (write in box)
18. University of Toronto has recently resumed some in-person classes. Are any of your classes in person? If so, how do you feel about attending in-person classes? (write in box)
19. Have you experienced, heard of, or witnessed any racism towards Asian Canadians during the pandemic? If so, what have you seen/heard/read? (write in box)
20. Do you usually get the seasonal flu vaccine?
    1. Yes (if yes, move to Question 43)
    2. No (if no, move to Question 44)
21. If yes, why? (Select all that apply)
    1. To avoid catching the flu
    2. My doctor recommends that I get it
    3. To avoid illness
    4. It is safe
    5. Worried about becoming seriously ill
    6. I always get the seasonal flu shot
    7. I live with people who are high risk
    8. I am high risk
    9. I am required to because of my job
    10. Other (write in below)
22. If no, why? (Select all that apply)
    1. It will not work
    2. Worried it will cause serious side effects
    3. Worried it will cause bothersome side effects
    4. Worried it will give me the flu
    5. It is not safe
    6. I am not at risk of catching the flu
    7. I don’t know where I would get it
    8. Other (write in below)
23. Did you get the seasonal flu vaccine in 2019?
    1. Yes
    2. No
    3. Can’t remember
24. Did you get the seasonal flu vaccine in 2020?
    1. Yes
    2. No
    3. Undecided
25. Have you received the COVID-19 vaccine?
    1. Yes, one shot (go to Question 27)
    2. Yes, two shots (go to Question 27)
    3. Yes, three or more shots (write in below) (go to Question 27)
    4. No (go to Question 26)
26. If no, why? (Select all that apply)
    1. It will not work
    2. Insufficient testing
    3. Worried it will cause serious side effects
    4. Worried it will cause bothersome side effects
    5. Worried it would give me COVID-19
    6. It is not safe
    7. I am not at risk of catching COVID-19
    8. I don’t know where I would get it
    9. Other (write in below)
27. If yes, why? (Select all that apply)
    1. To avoid catching COVID-19
    2. To avoid illness
    3. It is safe
    4. Worried about becoming seriously ill
    5. COVID-19 is deadlier than the seasonal flu
    6. I always get the seasonal flu shot
    7. I live with people who are high risk
    8. I am high risk
    9. I will be required to because of my job
    10. Other (write in below)
28. If your doctor or pharmacist recommended getting the COVID-19 vaccine would that encourage you to get it?
    1. Yes
    2. No
    3. Undecided
29. How do you feel about the possibility of vaccines being made mandatory at the federal/provincial/municipal level(s) in Canada? (write in box)
30. How do you feel about the recent plan to require vaccine passports for certain indoor activities in Ontario? How might this affect your life? (write in box)
31. University of Toronto recently announced a vaccine requirement for all students visiting the university campus How do you feel about this requirement? (write in box)
32. Do you have regular access to social media (e.g., Instagram, Facebook, Twitter, TikTok) as a news source?
    1. Yes (if yes, move to Questions 10-12)
    2. No (if no, move to Question 13)
33. In a typical week, how much time per DAY (in minutes) do you spend using social media for general use? (write in box)
34. In a typical week, how much time per DAY (in minutes) do you spend using social media as a source for COVID-19 health-related information?
35. How successful do you feel social media is in bringing clear, concise, and unbiased information about the COVID-19 outbreak?
    1. 7-point Likert scale (1: unsuccessful, 7: successful)
36. Do you have regular access to internet news sources (e.g., CBC, BBC, CNN, Globe and Mail) as a news source?)
    1. Yes (if yes, move to Questions 14-16)
    2. No (if no, move to Question 17)
37. In a typical week, how much time per DAY (in minutes) do you spend using internet news sources for general use? (write in box)
38. In a typical week, how much time per DAY (in minutes) do you spend using internet news sources as a source for COVID-19 health-related information? (write in box)
39. How successful do you feel internet news sources are in bringing clear, concise, and unbiased information about the COVID-19 outbreak?
    1. 7-point Likert scale (1: unsuccessful, 7: successful)
40. Do you have regular access to the radio as a news source?
    1. Yes (if yes, move to Questions 18-20)
    2. No (if no, move to Question 21)
41. In a typical week, how much time per DAY (in minutes) do you spend using the radio for general use? (write in box)
42. In a typical week, how much time per DAY (in minutes) do you spend using the radio as a source for COVID-19 health-related information? (write in box)
43. How successful do you feel the radio is in bringing clear, concise, and unbiased information about the COVID-19 outbreak?
    1. 7-point Likert scale (1: unsuccessful, 7: successful)
44. Do you have regular access to television as a news source?
    1. Yes (if yes, move to Questions 22-24)
    2. No (if no, move to Question 25)
45. In a typical week, how much time per DAY (in minutes) do you spend using television for general use? (write in box)
46. In a typical week, how much time per DAY (in minutes) do you spend using television as a source for COVID-19 health-related information? (write in box)
47. How successful do you feel television is in bringing clear, concise, and unbiased information about the COVID-19 outbreak?
    1. 7-point Likert scale (1: unsuccessful, 7: successful)
48. Do you have regular access to magazines as a news source?
    1. Yes (if yes, move to Questions 26-28)
    2. No (if no, move to Question 29)
49. In a typical week, how much time per DAY (in minutes) do you spend using magazines for general use? (write in box)
50. In a typical week, how much time per DAY (in minutes) do you spend using magazines as a source for COVID-19 health-related information? (write in box)
51. How successful do you feel magazines are in bringing clear, concise, and unbiased information about the COVID-19 outbreak?
    1. 7-point Likert scale (1: unsuccessful, 7: successful)
52. How much time does the media spend covering the COVID-19 outbreak?
    1. 7-point Likert scale (1: not enough, 7: too much)
53. Which form of media made you feel the most anxiety and fear about becoming infected with COVID-19?
    1. Social media
    2. Internet news source
    3. Radio
    4. Television
    5. Magazines
    6. Other (write in below)
54. Did you participate in the first round (March/April 2020), second round (June/July 2020), third round (September/October 2020), or fourth round (March/April 2021) of this survey? Select all that apply.
    1. First round (March/April 2020)
    2. Second round (June/July 2020)
    3. Third round (September/October 2020)
    4. Fourth round (March/April 2021)
    5. None of these
55. As part of our research we are looking for participants to be interviewed about their perceptions of COVID-19 over videoconferencing. All interviews will be confidential. If you are interested in receiving more information about the interview, please write your email into the box below and we will contact you. Providing your email does NOT mean you must participate.
56. Thank you for participating in our survey! If you would like to be entered into a draw for one of three $50 gift cards please enter your email below. Please press “Submit” below to complete the survey.

**Survey Guide Round 6: March/April 2022**

1. What is your age? (write in box)
2. To which gender identity do you most identify?
   1. Male
   2. Female
   3. Gender variant/non-conforming
   4. Prefer not to answer
   5. Prefer to self describe (write in box below)
3. How do you describe your ethnicity? (write in box)
4. What is your year of study at the University of Toronto?
   1. Undergraduate year 1
   2. Undergraduate year 2
   3. Undergraduate year 3
   4. Undergraduate year 4
   5. Undergraduate year 5
   6. Undergraduate year other
   7. Graduate
5. What is your program of study at the University of Toronto? (write in box)
6. What is the location of your permanent residence?
   1. Greater Toronto Area (GTA)
   2. Southwestern Ontario (other than the GTA)
   3. Northern Ontario
   4. Quebec
   5. Eastern Canadian provinces
   6. Western Canadian provinces
   7. Eastern Ontario
   8. Other (write in below)
7. What is your household income (if you support yourself) or your family’s annual household income, if they provide more than 50% of your income support (CAD)?
   1. Less than $24 999
   2. $25 000 to $49 999
   3. $50 000 to $74 999
   4. $75 000 to $99 999
   5. $100 000 to $124 999
   6. $125 000 to $149 999
   7. Greater than $150 000
8. Have you been personally affected by the current COVID-19 outbreak through the illness of yourself or an immediate family member?
   1. Yes
   2. No
9. How severe a threat is COVID-19?
   1. 7-point Likert scale (1: not severe, 7: very severe)
10. How anxious or fearful of acquiring COVID-19 are you after hearing or reading a news report updating the outbreak?
    1. 7-point Likert scale (1: not fearful/anxious, 7: very fearful/anxious)
11. Do you think you are at risk of contracting COVID-19?
    1. Yes
    2. No
12. Why do you think you are or are not at risk of contracting COVID-19? (write in box)
13. Has the news coverage of COVID-19 influenced your day-to-day behaviour? (Choose all that apply)
    1. No change
    2. Cancelled or changed travel plans
    3. I wear a mask some or all of the time
    4. I am washing my hands more
    5. I am using hand sanitizer
    6. I am social distancing
    7. I am self isolating
    8. I am cleaning more
    9. I have bought extra food/supplies
    10. I have gone to or plan to go to the doctor for help/advice
    11. I have gone to or plan to go to the hospital for help/advice
    12. I have called public health
    13. I have been tested for COVID-19
    14. I have chosen to get a COVID-19 test
    15. Other (write in box below)
14. What effects have the government-mandated social distancing and non-essential closures had on your life (employment, financial, social, etc.)? (write in box)
15. How are the social distancing rules and the pandemic in general affecting your mental health? (write in box)
16. What strategies, support systems, or programs for mental health or otherwise (economic, social) would you find beneficial at this time (what could the local, provincial, or federal government be doing to help you)? (write in box)
17. In the last two years have you noticed an increase in your use of substances (e.g., alcohol, cannabis, opioids, stimulants, sedatives, hallucinogens)? Why or why not? (write in box)
18. University of Toronto has recently resumed in-person classes. How do you feel about attending in-person classes? (write in box)
19. Have you experienced, heard of, or witnessed any racism towards Asian Canadians during the pandemic? If so, what have you seen/heard/read? (write in box)
20. Do you usually get the seasonal flu vaccine?
    1. Yes (if yes, move to Question 43)
    2. No (if no, move to Question 44)
21. If yes, why? (Select all that apply)
    1. To avoid catching the flu
    2. My doctor recommends that I get it
    3. To avoid illness
    4. It is safe
    5. Worried about becoming seriously ill
    6. I always get the seasonal flu shot
    7. I live with people who are high risk
    8. I am high risk
    9. I am required to because of my job
    10. Other (write in below)
22. If no, why? (Select all that apply)
    1. It will not work
    2. Worried it will cause serious side effects
    3. Worried it will cause bothersome side effects
    4. Worried it will give me the flu
    5. It is not safe
    6. I am not at risk of catching the flu
    7. I don’t know where I would get it
    8. Other (write in below)
23. Did you get the seasonal flu vaccine in 2019?
    1. Yes
    2. No
    3. Can’t remember
24. Did you get the seasonal flu vaccine in 2020?
    1. Yes
    2. No
    3. Undecided
25. Did you get the seasonal flu vaccine in 2021?
    1. Yes
    2. No
    3. Undecided
26. Have you received the COVID-19 vaccine?
    1. Yes, one shot (go to Question 27)
    2. Yes, two shots (go to Question 27)
    3. Yes, three shots (go to Question 27)
    4. Yes, four or more shots (write in below)
    5. No (go to Question 28)
27. If yes, why? (Select all that apply)
    1. To avoid catching COVID-19
    2. To avoid illness
    3. It is safe
    4. Worried about becoming seriously ill
    5. COVID-19 is deadlier than the seasonal flu
    6. I always get the seasonal flu shot
    7. I live with people who are high risk
    8. I am high risk
    9. I will be required to because of my job
    10. Other (write in below)
28. If no, why? (Select all that apply)
    1. It will not work
    2. Insufficient testing
    3. Worried it will cause serious side effects
    4. Worried it will cause bothersome side effects
    5. Worried it would give me COVID-19
    6. It is not safe
    7. I am not at risk of catching COVID-19
    8. I don’t know where I would get it
    9. Other (write in below)
29. If your doctor or pharmacist recommended getting the COVID-19 vaccine would that encourage you to get it?
    1. Yes
    2. No
    3. Undecided
30. How do you feel about the possibility of vaccines being made mandatory at the federal/provincial/municipal level(s) in Canada? (write in box)
31. How do you feel about the requirement for vaccine passports for certain indoor activities in Ontario that started last fall? How did this affect your life? (write in box)
32. How do you feel about University of Toronto requiring all students visiting the university campus to be vaccinated? How did this affect your life? (write in box)
33. What are your impressions of the recent Omicron variant? How do you feel about the Canadian/provincial governments’ response to it? (write in box)
34. Ontario has recently removed mask mandates for most indoor spaces. How do you feel about this decision? Will you continue to wear a mask? Why or why not? (write in box)
35. Do you have regular access to social media (e.g., Instagram, Facebook, Twitter, TikTok) as a news source?
    1. Yes (if yes, move to Questions 10-12)
    2. No (if no, move to Question 13)
36. In a typical week, how much time per DAY (in minutes) do you spend using social media for general use? (write in box)
37. In a typical week, how much time per DAY (in minutes) do you spend using social media as a source for COVID-19 health-related information?
38. How successful do you feel social media is in bringing clear, concise, and unbiased information about the COVID-19 outbreak?
    1. 7-point Likert scale (1: unsuccessful, 7: successful)
39. Do you have regular access to internet news sources (e.g., CBC, BBC, CNN, Globe and Mail) as a news source?)
    1. Yes (if yes, move to Questions 14-16)
    2. No (if no, move to Question 17)
40. In a typical week, how much time per DAY (in minutes) do you spend using internet news sources for general use? (write in box)
41. In a typical week, how much time per DAY (in minutes) do you spend using internet news sources as a source for COVID-19 health-related information? (write in box)
42. How successful do you feel internet news sources are in bringing clear, concise, and unbiased information about the COVID-19 outbreak?
    1. 7-point Likert scale (1: unsuccessful, 7: successful)
43. Do you have regular access to the radio as a news source?
    1. Yes (if yes, move to Questions 18-20)
    2. No (if no, move to Question 21)
44. In a typical week, how much time per DAY (in minutes) do you spend using the radio for general use? (write in box)
45. In a typical week, how much time per DAY (in minutes) do you spend using the radio as a source for COVID-19 health-related information? (write in box)
46. How successful do you feel the radio is in bringing clear, concise, and unbiased information about the COVID-19 outbreak?
    1. 7-point Likert scale (1: unsuccessful, 7: successful)
47. Do you have regular access to television as a news source?
    1. Yes (if yes, move to Questions 22-24)
    2. No (if no, move to Question 25)
48. In a typical week, how much time per DAY (in minutes) do you spend using television for general use? (write in box)
49. In a typical week, how much time per DAY (in minutes) do you spend using television as a source for COVID-19 health-related information? (write in box)
50. How successful do you feel television is in bringing clear, concise, and unbiased information about the COVID-19 outbreak?
    1. 7-point Likert scale (1: unsuccessful, 7: successful)
51. Do you have regular access to magazines as a news source?
    1. Yes (if yes, move to Questions 26-28)
    2. No (if no, move to Question 29)
52. In a typical week, how much time per DAY (in minutes) do you spend using magazines for general use? (write in box)
53. In a typical week, how much time per DAY (in minutes) do you spend using magazines as a source for COVID-19 health-related information? (write in box)
54. How successful do you feel magazines are in bringing clear, concise, and unbiased information about the COVID-19 outbreak?
    1. 7-point Likert scale (1: unsuccessful, 7: successful)
55. How much time does the media spend covering the COVID-19 outbreak?
    1. 7-point Likert scale (1: not enough, 7: too much)
56. Which form of media made you feel the most anxiety and fear about becoming infected with COVID-19?
    1. Social media
    2. Internet news source
    3. Radio
    4. Television
    5. Magazines
    6. Other (write in below)
57. Did you participate in the first round (March/April 2020), second round (June/July 2020), third round (September/October 2020), fourth round (March/April 2021), or fifth round (September October 2021) of this survey? Select all that apply.
    1. First round (March/April 2020)
    2. Second round (June/July 2020)
    3. Third round (September/October 2020)
    4. Fourth round (March/April 2021)
    5. Fifth round (September/October 2021)
    6. None of these
58. As part of our research we are looking for participants to be interviewed about their perceptions of COVID-19 over videoconferencing. All interviews will be confidential. If you are interested in receiving more information about the interview, please write your email into the box below and we will contact you. Providing your email does NOT mean you must participate.
59. Thank you for participating in our survey! If you would like to be entered into a draw for one of three $50 gift cards please enter your email below. Please press “Submit” below to complete the survey.

# INTERVIEW QUESTIONS

*Questions were added and removed as indicated.*

1. Where have you gotten information on COVID-19?
   1. Have you gone looking for information? If so where and why? If not, why not?
   2. Are you receiving it passively? Where are you getting it?
   3. If getting it on social media, are you looking it up or just unable to avoid it? How do you feel about the information you are getting on social media?
   4. Do you go looking on news sites, or just follow ones from social media?
   5. Your news/updates: are you getting them locally, from other provinces, Canada, around the world?
   6. Are you spending more/less/the same time seeking information about COVID than you were over the past year?
   7. [if they haven’t already spoken about vaccines] What kind of information have you seen about vaccines? Where are you getting your information about vaccines?
   8. What would you have liked to know that wasn’t available?
2. Are you concerned about COVID-19? Why or why not?
   1. How serious is this virus? What makes you think that? Has your perception of the seriousness changed over this past year(s)?
   2. Do you feel you are susceptible? – explain why or why not
   3. Have you had a COVID-19 test?
   4. Are you concerned about the variants? [if they haven’t brought this up so far] (removed Spring 2022)
   5. What was your impression of Omicron? Have you heard of BA.2? (added Spring 2022)
   6. Have you had a personal experience with COVID-19? Family members? Friends?
   7. When do you think this will end? (Be totally gone? Be less of a problem?)
3. Are you doing anything to avoid contracting COVID-19?
   1. Are you still practicing social distancing? And how is it affecting your life?
   2. Are you in a hotspot? What is your reaction to this (are you surprised that you are/aren’t, why)? (added March/April 2021)
   3. Is social distancing affecting your mental health? If so, how?
      1. Have you accessed any services during the pandemic, such as mental health services, food banks, or signed up for any benefit programs? Or EI?
      2. Did you use any of these before?
   4. Have you found that you’ve increased your use of alcohol or drugs including cannabis during this time? (added September/October 2020)
   5. Are you working right now? How has social distancing affected your work?
   6. Financial (if not brought up under previous questions)
      1. Where does your income come from?
      2. Does social distancing affect your ability to work? (next few weeks/months)
      3. Will this impact you for next year?
4. Do you think the government’s response to COVID-19 is adequate? What, if anything, should be done differently?
   1. If the answer is “more supports for people”: what kind of supports would be beneficial?
5. What do you think of how your university has been handling things lately? How was the past semester for you (with the return to in-person teaching)? (Spring 2022)
6. Are you aware of any racist incidents toward the Asian-Canadian and/or Chinese communities linked to the COVID-19 pandemic?
7. If/when there is a vaccine available for COVID-19, would you get it? (added June/July 2020, removed in March/April 2021)
8. Have you received your COVID-19 vaccine and/or do you plan to receive it when you become eligible? (added March/April 2021)
   1. If not, what are your specific concerns?
   2. Which of the available vaccines would you be willing to get?
   3. Have you had your booster? (added Spring 2022)
9. What do you miss most about the pre-pandemic period? (added March/April 2021)
10. What are you looking forward to? (added March/April 2021)
11. Is there anything else you wanted to tell us that we haven’t asked you?
