# Supplementary material for: Mixed-methods study of university students’ perceptions of COVID-19 and media consumption from March 2020 –April 2022
Source: PLOS Glob Public Health. 2024 Jul 17;4(7):e0003251. doi: 10.1371/journal.pgph.0003251 (PMC11253943; doi:10.1371/journal.pgph.0003251)
Supplement: S2 Text — Likelihood ratio tests of demographic predictors for judgement of severity of COVID-19, susceptibility to COVID-19, and adoption of new health behaviours; Table B. Binary logistic regression results for judgement of severity of COVID-19, susceptibility to COVID-19, Poisson regression results for new health behaviours; Table C. Likelihood ratio tests of demographic predictors for feelings of anxiety/fear after hearing/reading a COVID-19 news report; Table D. Logistic regression analysis results for anxiety/fear after hearing/reading a COVID-19 news report; Table E. Multinomial logistic regression results regarding the forms of media that made participants feel most anxiety/fear about becoming infected with COVID-19; Table F. Binary logistic regression for judgement of severity of COVID-19; Table G. Interview participants’ demographics. (DOCX) [file pgph.0003251.s002.docx]

**Supporting Materials 2: Results Tables**

Table A. Likelihood ratio tests of demographic predictors for judgement of severity of COVID-19, susceptibility to COVID-19, and adoption of new health behaviours

| **Predictor** | $\boldsymbol{\chi}^{\boldsymbol{2}}$ | $\boldsymbol{df}$ | ***p*-value** |
| --- | --- | --- | --- |
| *Main effects of predictors on judgement of severity of COVID-19* | | | |
| Age | 0.592 | 1 | 0.442 |
| Gender^a^ | 72.276 | 1 | < 0.001 |
| Program | 1.350 | 1 | 0.245 |
| Income | 1.438 | 2 | 0.487 |
| Time^a^ | 93.900 | 5 | < 0.001 |
| *Main effects of predictors on judgement of susceptibility to COVID-19* | | | |
| Age^a^ | 128.435 | 1 | < 0.001 |
| Gender^a^ | 17.943 | 1 | < 0.001 |
| Program | 2.560 | 1 | 0.110 |
| Income | 3.650 | 2 | 0.161 |
| Time^a^ | 28.302 | 5 | < 0.001 |
| *Main effects of predictors for new health behaviours* | | | |
| Age | 0.096 | 1 | 0.756 |
| Gender^a^ | 44.873 | 1 | < 0.001 |
| Program | 0.002 | 1 | 0.965 |
| Income | 1.879 | 2 | 0.391 |
| Time^a^ | 48.196 | 5 | < 0.001 |

1. $p$< 0.05.

Table B. Binary logistic regression results for judgement of severity of COVID-19, susceptibility to COVID-19, Poisson regression results for new health behaviours

| **Predictor** | $\hat{\boldsymbol{\beta}}$ | $\boldsymbol{SE}$**(**$\hat{\boldsymbol{\beta}}$**)** | **Wald** | ***p*-value** | **Exp (**$\hat{\boldsymbol{\beta}}$**)** |
| --- | --- | --- | --- | --- | --- |
| *Binary logistic regression for judgement of severity of COVID-19* | | | | | |
| (Intercept) | 3.065 | 0.613 | 4.997 | < 0.001 | 21.431 |
| Age | -0.018 | 0.023 | -0.774 | 0.439 | 0.982 |
| Gender (Female)^c^ | 0.936 | 0.108 | 8.702 | < 0.001 | 2.550 |
| Program (Non-Health Studies) | -0.219 | 0.193 | -1.137 | 0.255 | 0.803 |
| Income (Low Level)^a^ | 0.187 | 0.157 | 1.190 | 0.234 | 1.206 |
| Income (Middle Level)^b^ | 0.172 | 0.164 | 1.049 | 0.294 | 1.187 |
| Time 2 (Summer 2020) | -0.586 | 0.318 | -1.844 | 0.065 | 0.556 |
| Time 3 (Fall 2020)^c^ | -0.623 | 0.269 | -2.314 | 0.021 | 0.537 |
| Time 4 (Spring 2021)^c^ | -0.749 | 0.271 | -2.761 | 0.006 | 0.473 |
| Time 5 (Fall 2021)^c^ | -0.932 | 0.264 | -3.533 | < 0.001 | 0.394 |
| Time 6 (Spring 2022)^c^ | -1.782 | 0.260 | -6.842 | < 0.001 | 0.168 |
| *Binary logistic regression for judgement of susceptibility to COVID-19* | | | | | |
| (Intercept) | -2.854 | 0.351 | -8.133 | < 0.001 | 0.057 |
| Age^c^ | 0.155 | 0.0141 | 10.948 | < 0.001 | 1.167 |
| Gender (Female)^c^ | 0.291 | 0.0685 | 4.240 | < 0.001 | 1.337 |
| Program (Non-Health Studies) | -0.164 | 0.103 | -1.592 | 0.111 | 0.849 |
| Income (Low Level)^a^ | -0.182 | 0.096 | -1.904 | 0.057 | 0.834 |
| Income (Middle Level)^b^ | -0.146 | 0.0995 | -1.464 | 0.143 | 0.864 |
| Time 2 (Summer 2020) | 0.0869 | 0.135 | 0.6444 | 0.519 | 1.091 |
| Time 3 (Fall 2020) | 0.188 | 0.108 | 1.740 | 0.082 | 1.207 |
| Time 4 (Spring 2021) | 0.107 | 0.111 | 0.962 | 0.336 | 1.1126 |
| Time 5 (Fall 2021) | -0.155 | 0.108 | -1.430 | 0.153 | 0.857 |
| Time 6 (Spring 2022)^c^ | 0.322 | 0.119 | 2.697 | 0.007 | 1.379 |
| *Poisson regression results for new health behaviours* | | | | | |
| (Intercept) | 0.552 | 0.109 | 5.073 | < 0.001 | 1.736 |
| Age | 0.001 | 0.0049 | 0.310 | 0.756 | 1.001 |
| Gender (Female)^c^ | 0.153 | 0.0239 | 6.609 | < 0.001 | 1.165 |
| Program (Non-Health Studies) | -0.001 | 0.032 | -0.044 | 0.965 | 0.999 |
| Income (Low Level)^a^ | -0.035 | 0.031 | -1.127 | 0.260 | 0.966 |
| Income (Middle Level)^b^ | -0.012 | 0.032 | -0.385 | 0.700 | 0.988 |
| Time 2 (Summer 2020)^c^ | 0.176 | 0.044 | 4.017 | < 0.001 | 1.193 |
| Time 3 (Fall 2020)^c^ | 0.191 | 0.036 | 5.312 | < 0.001 | 1.211 |
| Time 4 (Spring 2021)^c^ | 0.186 | 0.037 | 5.048 | < 0.001 | 1.205 |
| Time 5 (Fall 2021)^c^ | 0.127 | 0.037 | 3.480 | < 0.001 | 1.136 |
| Time 6 (Spring 2022)^c^ | 0.048 | 0.040 | 1.188 | 0.235 | 1.049 |

1. <$24,999-$74,999/annum
2. <$75,000-$149,999/annum
3. $p$< 0.05.

Table C. Likelihood ratio tests of demographic predictors for feelings of anxiety/fear after hearing/reading a COVID-19 news report

| *Main effects of predictors for anxiety/fear after COVID-19 report* | | | |
| --- | --- | --- | --- |
| Age | 1.068 | 1 | 0.301 |
| Gender^a^ | 185.479 | 1 | < 0.001 |
| Program | 0.052 | 1 | 0.819 |
| Income^a^ | 9.651 | 2 | 0.008 |
| Time^a^ | 49.035 | 5 | < 0.001 |

Table D. Logistic regression analysis results for anxiety/fear after hearing/reading a COVID-19 news report

| *Binary logistic regression results for anxiety/fear after COVID-19 report* | | | | | |
| --- | --- | --- | --- | --- | --- |
| (Intercept) | -0.093 | 0.382 | -0.243 | 0.808 | 0.912 |
| Age | 0.016 | 0.015 | 1.031 | 0.303 | 1.016 |
| Gender (Female)^c^ | 0.993 | 0.072 | 13.749 | < 0.001 | 2.698 |
| Program (Non-Health Studies) | -0.026 | 0.115 | -0.228 | 0.820 | 0.974 |
| Income (Low Level)^a, c^ | 0.315 | 0.104 | 3.042 | 0.002 | 1.371 |
| Income (Middle Level)^b^ | 0.197 | 0.108 | 1.831 | 0.067 | 1.217 |
| Time 2 (Summer 2020) | -0.291 | 0.153 | -1.900 | 0.057 | 0.747 |
| Time 3 (Fall 2020) | -0.006 | 0.127 | -0.048 | 0.962 | 0.994 |
| Time 4 (Spring 2021) | 0.201 | 0.133 | 1.512 | 0.130 | 1.223 |
| Time 5 (Fall 2021) | -0.127 | 0.127 | -0.999 | 0.317 | 0.881 |
| Time 6 (Spring 2022)^c^ | -0.542 | 0.133 | -4.079 | < 0.001 | 0.581 |

Table E. Multinomial logistic regression results regarding the forms of media that made participants feel most anxiety/fear about becoming infected with COVID-19

|  | $\hat{\boldsymbol{\beta}}$ | $\boldsymbol{SE}$**(**$\hat{\boldsymbol{\beta}}$**)** | **Wald** | ***p*-value** | **Exp (**$\hat{\boldsymbol{\beta}}$**)** |
| --- | --- | --- | --- | --- | --- |
| **Social Media** |  |  |  |  |  |
| (Intercept) | 5.438 | 0.7378 | 7.378 | < 0.001 | 229.948 |
| Age^c^ | -0.120 | 0.027 | -4.522 | < 0.001 | 0.887 |
| Gender (Female)^c^ | 0.639 | 0.140 | 4.564 | < 0.001 | 1.895 |
| Program (Non-Health Studies)^c^ | -0.807 | 0.277 | -2.914 | 0.005 | 0.446 |
| Income (Low Level)^a^ | -0.092 | 0.224 | -0.413 | 0.680 | 0.912 |
| Income (Middle Level)^b^ | -0.095 | 0.234 | -0.406 | 0.685 | 0.909 |
| Time 2 (Summer 2020) | -0.251 | 0.340 | -0.740 | 0.459 | 0.778 |
| Time 3 (Fall 2020) | -0.529 | 0.275 | -1.921 | 0.055 | 0.589 |
| Time 4 (Spring 2021) | -0.586 | 0.277 | -2.114 | 0.035 | 0.557 |
| Time 5 (Fall 2021) | -0.414 | 0.278 | -1.486 | 0.137 | 0.661 |
| Time 6 (Spring 2022) | -0.411 | 0.294 | -1.398 | 0.162 | 0.663 |
| **Internet** |  |  |  |  |  |
| (Intercept) | 4.876 | 0.763 | 6.388 | < 0.001 | 131.114 |
| Age^c^ | -0.116 | 0.028 | -4.180 | < 0.001 | 0.891 |
| Gender (Female)^c^ | 0.628 | 0.146 | 4.301 | < 0.001 | 1.873 |
| Program (Non-Health Studies)^c^ | -0.970 | 0.282 | -3.445 | < 0.001 | 0.379 |
| Income (Low Level)^a^ | -0.157 | 0.231 | -0.681 | 0.496 | 0.854 |
| Income (Middle Level)^b^ | -0.088 | 0.241 | -0.364 | 0.716 | 0.916 |
| Time 2 (Summer 2020) | -0.213 | 0.352 | -0.605 | 0.545 | 0.809 |
| Time 3 (Fall 2020) | -0.248 | 0.284 | -0.873 | 0.383 | 0.781 |
| Time 4 (Spring 2021) | -0.5058 | 0.287 | -1.761 | 0.078 | 0.603 |
| Time 5 (Fall 2021) | -0.204 | 0.287 | -0.710 | 0.478 | 0.816 |
| Time 6 (Spring 2022) | -0.316 | 0.304 | -1.039 | 0.299 | 0.729 |
| **TV** |  |  |  |  |  |
| (Intercept) | 5.340 | 0.776 | 6.882 | < 0.001 | 208.427 |
| Age^c^ | -0.138 | 0.028 | -4.874 | < 0.001 | 0.871 |
| Gender (Female)^c^ | 0.696 | 0.149 | 4.681 | < 0.001 | 2.005 |
| Program (Non-Health Studies)^c^ | -0.894 | 0.285 | -3.138 | 0.0021 | 0.409 |
| Income (Low Level)^a^ | -0.276 | 0.233 | -1.187 | 0.235 | 0.759 |
| Income (Middle Level)^b^ | -0.105 | 0.243 | -0.431 | 0.667 | 0.907 |
| Time 2 (Summer 2020) | -0.263 | 0.351 | -0.749 | 0.454 | 0.769 |
| Time 3 (Fall 2020) | -0.486 | 0.284 | -1.710 | 0.087 | 0.615 |
| Time 4 (Spring 2021) | -0.615 | 0.287 | -2.144 | 0.032 | 0.541 |
| Time 5 (Fall 2021) | -0.457 | 0.288 | -1.587 | 0.113 | 0.633 |
| Time 6 (Spring 2022) | -0.609 | 0.306 | -1.992 | 0.046 | 0.544 |

Table F. Binary logistic regression for judgement of severity of COVID-19

| **Predictor** | $\hat{\boldsymbol{\beta}}$ | $\boldsymbol{SE}$**(**$\hat{\boldsymbol{\beta}}$**)** | **Wald** | ***p*-value** | **Exp (**$\hat{\boldsymbol{\beta}}$**)** |
| --- | --- | --- | --- | --- | --- |
| *Severity of COVID-19* | | | | | |
| (Intercept) | 0.949 | 0.171 | 5.542 | < 0.001 | 2.583 |
| Fear of acquiring COVID^a^ | 2.131 | 0.115 | 18.602 | < 0.001 | 8.425 |
| Type of Media (Social Media)^a^ | 0.408 | 0.193 | 2.119 | 0.034 | 1.504 |
| Type of Media (Internet) | 0.267 | 0.202 | 1.326 | 0.185 | 1.307 |
| Type of Media (TV) | 0.203 | 0.203 | 1.001 | 0.317 | 1.226 |
| *Susceptibility to COVID-19* | | | | | |
| (Intercept) | -0.203 | 0.127 | -1.596 | 0.111 | 0.816 |
| Fear of acquiring COVID^a^ | 0.729 | 0.066 | 11.003 | < 0.001 | 2.073 |
| Type of Media (Social Media) | -0.043 | 0.133 | -0.326 | 0.745 | 0.958 |
| Type of Media (Internet) | 0.055 | 0.137 | 0.398 | 0.690 | 1.056 |
| Type of Media (TV) | -0.069 | 0.138 | -0.498 | 0.618 | 0.933 |

1. $p$< 0.05.

Table G. Interview participants’ demographics

|  | Spring 2020  n (%) | Summer 2020  n (%) | Fall 2020  n (%) | Spring 2021  n (%) | Fall 2021  n (%) | Spring 2022  n (%) |
| --- | --- | --- | --- | --- | --- | --- |
| Total | 27 | 17 | 17 | 15 | 19 | 18 |
| *Sex* | | | | | | |
| Male | 8 (29.6) | 5 (29.4) | 6 (35.3) | 6 (40.0) | 6 (31.6) | 7 (38.9) |
| Female | 18 (66.7) | 10 (58.8) | 9 (52.9) | 8 (53.3) | 11 (57.9) | 10 (55.6) |
| Gender  Variant | 1 (3.7) | 2 (11.8) | 2 (11.8) | 1 (6.7) | 2 (10.5) | 1 (5.6) |
| *Healthcare field* | | | | | | |
| Yes | 3 (11.1) | 2 (11.8) | 2 (11.8) | 1 (6.7) | 0 | 0 |
| No | 24 (88.9) | 15 (88.2) | 15 (88.2) | 14 (93.3) | 19 (100.0) | 18 (100.0) |
| *Household Income* | | | | | | |
| <$24,999 | 4 (14.8) | 4 (23.5) | 3 (17.6) | 2 (13.3) | 4 (21.1) | 5 (27.8) |
| $25,000 - $49,999 | 5 (18.5) | 4 (23.5) | 3 (17.6) | 4 (26.7) | 3 (15.8) | 2 (11.1) |
| $50,000 - $74,999 | 6 (22.2) | 2 (11.8) | 4 (23.5) | 1 (6.7) | 2 (10.5) | 1 (5.6) |
| $75,000-$99,000 | 3 (11.1) | 4 (23.5) | 2 (11.8) | 4 (26.7) | 2 (10.5) | 3 (16.7) |
| $100,000-$124,999 | 2 (7.4) | 2 (11.8) | 3 (17.6) | 3 (20.0) | 3 (15.8) | 2 (11.1) |
| $125,000-$149,999 | 3 (11.1) | 0 | 0 | 0 | 2 (10.5) | 3 (16.7) |
| $150,000 + | 3 (11.1) | 1 (5.9) | 2 (11.8) | 1 (6.7) | 3 (15.8) | 2 (11.1) |
| No response | 1 (3.7) | 0 | 0 | 0 | 0 | 0 |
