# Supplementary material for: Mixed-methods study of university students’ perceptions of COVID-19 and media consumption from March 2020 –April 2022
Source: PLOS Glob Public Health. 2024 Jul 17;4(7):e0003251. doi: 10.1371/journal.pgph.0003251 (PMC11253943; doi:10.1371/journal.pgph.0003251)
Supplement: Attachment — Submitted filename: Response to Reviewers.docx [file pgph.0003251.s003.docx]

April 29, 2024

Dear Dr. Brault,

Thank you so much for your email of April 4, 2024. My co-authors and I have revised our manuscript in response to the helpful comments provided by yourself and the reviewers. All four co-authors have reviewed and agreed to the following changes. The changes made are indicated in italicized text and all line numbers refer to the manuscript marked with track changes.

**Editor comments**:

- The reviewers and I found the manuscript to be well-written, and feel that it will make a useful contribution to the growing literature on young adult experiences in the context of COVID-19. However, there are several places where additional methodological clarity would improve the manuscript.

*Thank you for your positive assessment of the manuscript.*

- Specifically, both reviewers and I have several questions regarding the methods and design. The approach used to integrate qualitative and quantitative findings needs further description.

*In response to the Editor and Reviewers’ comments we have added details regarding the data collection, cleaning, and analysis procedures. The Qualitative Methodology heading has been changed to “Qualitative and integrative mixed-methods methodology” to signpost this.*

- In addition, I was unclear as to why the qualitative results were only partially presented in the body of the results, with the bulk of the material included in a supplemental file. Given that the journal does not have a specific word count, and it make it harder on the reader, I would encourage the authors to include the qualitative findings in the main manuscript. If there are additional quotes or secondary materials, then these could be placed in a supplemental file. Further, qualitative quotes should not be presented in the Discussion. They should be presented and analyzed in the results, with the other data.

*Thank you for the encouragement to include these in the manuscript body. Tables S9 and S10 have been moved from the Supplementary Materials 2 file and are now Tables 8 and 9 in the main text. The quotations that were previously highlighted in the discussion have either been moved into the Results section or deleted from the main text.*

**Reviewer #1**: The aim of the manuscript is to investigate the perceptions and behaviors of university students regarding the COVID-19 pandemic over a period of time through a mixed method approach. The study aims to explore how various factors, including gender, age, media consumption, and government mandates, influence students’ perceptions of severity, susceptibility, adoption of health behaviors, and feeling of anxiety/fear. It also provides insights into the sources of COVID-19 information preferred by university students and how these sources impact their perceptions and behaviors.


INTRODUCTION:

The paper suggests using a mixed-methods approach for understanding changing perceptions and behaviors. It could be clearer on why both quantitative and qualitative data are needed and how they work together to tackle the research questions.

*The purpose of this paper is to explore how perceptions about the pandemic, associated health behaviours, and engagement with media sources changed over time. A mixed method approach is necessary as these questions cover areas that are best served by qualitative and quantitative data. For example, frequency data on the use of different media types is best measured by surveys, but the nuanced ways these media sources were used to change behaviours or perceptions is best understood though interviews. Similarly, while information on common health behaviours and frequency changes through time can be gathered by surveys, understanding the motivation for these changes is accessed through open-ended interview questions. We have added text to this effect (lines 71-73, 110-113, 233-237) and added two new references [16 and 17] to underline the importance of the mixed-methods approach.*

*16.	Dawadi S, Shrestha S, Giri RA. Mixed-Methods Research: A Discussion on its Types, Challenges, and Criticisms. Journal of Practical Studies in Education. 2021;2: 25–36. doi:10.46809/jpse.v2i2.20*

*17.	Creswell JW, Plano VL. Designing and Conducting Mixed Methods Research. 3rd ed. SAGE Publications Inc.; 2017.*

METHODS:

Design:
The paper describes the mixed methods design used in the study, which appears to be concurrent, with qualitative and quantitative data collected simultaneously. A visual display of the overall design highlighting integration, which could enhance clarity for readers.

*We have created a timeline figure (new Figure 1) which shows the six periods of survey and interview with key events (e.g., declaration of pandemic, mask mandates in the province) affecting the study sample.*

Subjects/Participants:

The sampling and recruitment is adequately described. Please give an estimation of the response rate. Are there any anonymous identifiers identifying participants who answered multiple rounds of the surveys.

*There is no estimated response rate as we do not have information regarding how many individuals viewed the survey and then decided whether or not to take the survey. The survey link was posted on Anthropology Department website, shared on social media sites (Twitter, Facebook), and shared with colleagues who could choose whether or not to share the link with their students. There are no anonymous identifiers identifying participants who answered multiple rounds of the survey. We have added this to the limitations (lines 366-369)..*

Methodological Rigor:

There is no discussion of efforts to ensure data quality, such as data cleaning procedures or inter-rater reliability checks, member checking, triangulation, or flexibility. Without mentioning these aspects, it’s challenging to assess the rigor of the quantitative methods employed.

*We note in the manuscript that “participants who reported ‘implausible values’” for media source use were removed (following Riehm et al., 2020). We added in the Quantitative methodology that surveys with <75% questions answered were removed from the final sample (lines 183-185). We added that authors MM and AH coded and crosschecked the demographic data following data cleaning (lines 184-185). We adjusted lines 262 and 263 in the Results to note “Of the 5124 total surveys* ***in the cleaned sample****…”. We have added information regarding the representativeness of the sample in reference to the total student population (lines 204-209). We have added details to the Qualitative and Integrative Mixed-Methods Methodology section to note that the interview and survey guides were designed with triangulation in mind (lines 237-241). We have also added that author MM returned to the transcripts again following the first round of analyses to look for contextual details (lines 246-248).*

RESULTS:

All components of the study findings have been clearly presented, including both qualitative and quantitative results. The integration of findings from both methods has been effectively demonstrated, such as through joint displays.

*Thank you to the Reviewer for this positive assessment of the results presentation.*

DISCUSSION:

This section effectively incorporates the implications of the integrated findings, provides synthesis and interpretation of the results in the context of existing literature and theoretical/conceptual frameworks, and includes a subsection on limitations.

Strengths:
Effectively synthesizes the findings, drawing connections between the results and existing literature.
The integration of qualitative interview data enriches the discussion, providing insights into participants’ experiences and perceptions.
The discussion acknowledges the limitations of the study, such as the convenience sample and the potential lack of generalizability.

*Thank you again for this summary of the strengths of the manuscript. In response to the Editor and Reviewer 2’s points about adding details to the Limitations section we have added that participants were not linked across the survey iterations.* *In response to the Editor’s point about moving the quotations into the Results section, we have moved all quotations to the Results section.*

Suggestions for Improvement:
Explore potential practical applications or recommendations

*We have expanded the final discussion paragraph (lines 354-360), including the following recommendation: “Consistent, real-time, clearly cited messaging with localized resources is necessary to engender trust and encourage action.”*

Providing links between the study’s theoretical/conceptual framework and the interpretation of findings could strengthen the theoretical grounding of the discussion.

*We agree and have added signposts regarding the Health Belief Model (lines 573-576, 661-662, 780-782, 825-826) throughout the discussion.*

Consider discussing potential avenues for future research or areas where further investigation is needed to build upon the current findings.

*We have reorganized the Strengths and Limitations paragraph to be entitled Strengths, Limitations, and Future Research. We note that more mixed-methods research in Canada and globally should be conducted to get a better sense of international variability (lines 884-888). We suggest that interviewing a cohort of the interviewees from this research in future with targeted questions regarding health behaviours could provide insights on long term lessons (lines 888-890).*

**Reviewer #2**: Dear Editor,

Thank you for providing the opportunity to review this work. The authors have conducted a study to describe the changes in risk perception and factors associated with it. They also describe changes in preventive practices over time throughout the study, as well as the influence of exposure to communication media. The manuscript is very well written and clearly divided into sections and subsections, which facilitate easy reading despite the lengthy list of results presented by the authors. I have no major comments; however, there are some minor points that need clarification or improvement before publication.

*Thank you to the reviewer for this, particularly the comment regarding the facilitation of easy reading!*

Study design: The authors need to describe the study design clearly. They mention that they conducted 6 surveys; however, it is not clear whether there are participants who could have participated 2 or more times. In such a case, it could be a cohort study requiring adjustment for repeated measures by time and individual. Alternatively, if they were unable to confirm the identity of all participants, this should be addressed as a limitation. Lastly, if it was ultimately a serial cross-sectional study, this should be clarified.

*While we know that some participants completed multiple surveys (as they sometimes indicated this in their free-form answers) we could not link these individuals between surveys. We have added this to the limitations section (lines 866-879).*

Population and sample: The authors indicate that the sample was convenience-based. It would be possible to perform a sensitivity analysis using age, sex, faculty, and other variables with which we can evaluate the university in general versus the sample to determine how representative the sample is, thereby understanding the effect of this. Perhaps it would be interesting to conduct a post-stratification analysis.

*We have added the following text to the Methods section to clarify*: *Since the student population of the University of Toronto is large (over 97,000) [21], the finite population correction [22] is ignored for evaluating whether this study’s convenience sample size is representative. The required sample size is at least 385 cases to estimate population proportions within 5% margin of error with 95% confidence of the true proportion. The study’s convenience sample sizes in each round of surveys meet this criterion (lines 204-209).*

External validity: In addition to the convenience sample, the authors conducted the study with a specific population of university students. This reduces the external validity of their results and should be further detailed when making comparisons with other studies and in the limitations section.

*We have revisited each place in the manuscript where a comparison is being made to clarify whether the other studies are focusing upon young adults, university students, and/or nationally representative groups. For instance, we removed the text “young adult and/or” at line 64 to make it clear that this manuscript was focused upon university students. At lines 819-820 we added that the study being mentioned is “nationally representative” and on line 641 we note that González-Castro et al.’s study focused upon “Spanish adults.” In the Strengths and Limitations section we adjusted the wording to say “university student groups or broader age or international contexts” (lines 864).*

Additional figure: It would be interesting to include a figure depicting the curve of cases and deaths associated with the region or country, with added lines or areas delineating the collection periods. Also, indicating important periods of the pandemic such as lockdown policies or mandatory mask usage would provide context for the pandemic in the study area.

*Reviewer 1 also mentioned such a figure, and in response we created the new Figure 1 which shows the periods of survey and interview collection. We elected not to overlay this with any lockdown dates on case/death curves as the piecemeal lockdowns that occurred in the province of Ontario were scattered, different for various health regions, and occurred off and on for weeks at a time.*

Finally, as a suggestion, it would be interesting to see Directed Acyclic Graphs (DAGs) for different outcomes and interrelated factors. It would also be interesting to see a unified DAG following the theoretical framework that the authors employed for the study.

*Thank you to the reviewer for this suggestion. We have added Figure 6, an adaptation of Rosenstock’s (1974) visualization of the Health Belief Model. This figure shows a key finding of the manuscript, that individuals’ perceptions influence their engagement and perception of the cues to action (i.e., media coverage of COVID-19).*

Additionally, discussing whether there is a way to unify all the results and factors would be beneficial to identify single points of public health intervention that can influence multiple outcomes.

*We have added a recommendation that public health communicators partner with universities and colleges to increase dissemination. We also summarize the results in that “consistent, real-time, clearly cited messaging with localized resources” is what the participants identified as desirable (lines 854-860).*
