# Supplementary material for: Mixed-methods study of university students’ perceptions of COVID-19 and media consumption from March 2020 –April 2022
Source: PLOS Glob Public Health. 2024 Jul 17;4(7):e0003251. doi: 10.1371/journal.pgph.0003251 (PMC11253943; doi:10.1371/journal.pgph.0003251)
Supplement: Attachment — Submitted filename: Response to Reviewers_R1.docx [file pgph.0003251.s004.docx]

**Response to Reviewers**

June 17, 2024

Dear Dr. Brault and the Reviewers:

Thank you for the opportunity to revise our manuscript (PGPH-D-24-00108R1). We have made the following changes (indicated in italics) in response to the comments provided. All four co-authors have seen and approved the revisions. NB: the line numbers referenced refer to the version of the manuscript with track changes.

Editor comments:

- The reviewers and I appreciate the authors' responsive to the previous comments, and feel that the manuscript is nearly ready to be accepted.
- However, there are a few areas requiring revision before this can be accepted (as no edits other than copy-editing are allowed once the manuscript is accepted).
- Please address Reviewer 2's questions concerning the selection model, and comments regarding Table and DAG formatting for consistency and clarity.

*Thank you for the positive assessment of our work. We appreciate Reviewer 2’s comments and have addressed them all, as detailed below.*

Reviewer #2: Thank you for the opportunity to review this work again. The authors have provided additional information and details that significantly enhance their work. They have clearly addressed all my questions and incorporated the necessary information into the text. I have aditional suggestions to further improve their work:

*Thank you for this positive assessment – we appreciated the initial comments provided and agree that the work was enhanced through their inclusion.*

1. In the methods section, include a description of the selection model and how the performance between the models was evaluated. Justify the inclusion of the variables in the model and explain why sociodemographic factors were not included in the binary logistic regression for assessing the severity of COVID-19. If a strategy was used for model selection, it is important to evaluate and show the changes in the coefficients between the saturated model and the final model.

*We did not undertake model selection for this research. We are following the scholarship of Alan Agresti, whose work on categorical data analysis does not suggest doing model selection by removing variables that are not statistically significant (i.e., arriving at a parsimonious model) if that is not in the scope of the research questions. For that reason, we included all demographic variables in all the logistic regression calculations. Demographic factors (i.e., participants’ age, gender, program of study, and income level) were included as factors in the binary logistic regression for assessing participants’ perceptions of the severity of COVID-19. These results are available in detail in Supporting Materials 2 (Table S1) and described on lines 283-289 and we note on line 285 that gender (p < 0.001) was predictive in participants’ perceptions of the severity of COVID-19.*

2. In Table 3, use consistent labels for the surveys (e.g., Survey 1, Survey 2, ... Survey 6) rather than seasonal terms like Fall and Spring. Standardize the terms in the manuscript to avoid misunderstandings.

*Thank you for this comment. We adjusted the labels for the surveys in Table 3 and 5. We also went through the manuscript to check for consistency, which resulted in the addition of the survey numbers in the Quantitative methodology section (lines 174-177), the first paragraph of the Results (lines 243-245), lines 388-390, in all cells of Table 10, and in the qualitative results presentation (between lines 509 and 574).*

3. Ensure that Tables 3, 4, and 7 use the same format, including the categories the frequency and percentage, to clearly show the direction of changes over time and the differences.

*We have taken the supporting summary statistics (previously Table S6 in Supporting Materials 2) and added them to Table 4 in the main text.*

*The results displayed in Table 7 draw upon the data displayed in Table 2 (Survey results for judgement of severity of COVID-19, judgement of personal susceptibility, and feelings of anxiety/fear after hearing/reading a news report regarding COVID-19) and in Table 5 (Participants’ judgements of which media source made them feel the most anxiety/fear about becoming infected with COVID-19); these tables display the requested categories with frequency and percentage. We have moved the table of Participants’ reported time using each media source (previously Table S5 in Supporting Materials 2) to become the new Table 7 in the main manuscript, such that all information used to calculate the results displayed in Table 7 (now Table 8) are in the main manuscript. To guide the reader to these, we have added a note below Table 8 that indicates the frequency data are available in Tables 2 and 5.*

4. For the DAG figures, use the following reference to standardize the symbols: Digitale JC, Martin JN, Glymour MM. Tutorial on directed acyclic graphs. J Clin Epidemiol. 2022 Feb;142:264-267. doi: 10.1016/j.jclinepi.2021.08.001. Epub 2021 Aug 8. PMID: 34371103; PMCID: PMC8821727.

*Thank you for providing the reference. We were unsure what the reviewer meant by standardizing the symbols, so we elected to simplify our figure to resemble those outlined by Digitale et al. and to use arrows of the same width to ensure we were not implying stronger relationships being associated with any arrow size. The revised figure is the newly uploaded Figure 6.*
